# Comprehensive review and expanding the genetic landscape of Cornelia-de-Lange spectrum: insights from novel mutations and skin biopsy in exome-negative cases

**DOI:** 10.1186/s12920-024-01798-7

**Published:** 2024-01-12

**Authors:** Sahand Tehrani Fateh, Nadia Mohammad Zadeh, Shadab Salehpour, Farzad Hashemi-Gorji, Ashkan Omidi, Hossein Sadeghi, Reza Mirfakhraie, Parinaz Moghimi, Sepideh Keyvanfar, Sepideh Mohammadi Sarvaleh, Mohammad Miryounesi, Mohammad-Reza Ghasemi

**Affiliations:** 1 Center for Comprehensive Genetic Services, Shahid Beheshti University of Medical Sciences, Tehran, Iran; 2 School of Medicine, Tehran University of Medical Sciences (TUMS), Tehran, Iran; 3 School of Medicine, Islamic Azad University Tehran Medical sciences, Tehran, Iran; 4 Department of Pediatrics, Clinical Research Development Unit, Loghman Hakim Hospital, Shahid Beheshti University of Medical Sciences, Tehran, Iran; 5 Genomic Research Center, Shahid Beheshti University of Medical Sciences, Tehran, Iran; 6 Department of Medical Genetics, Faculty of Medicine, Shahid Beheshti University of Medical Sciences, Tehran, Iran

**Keywords:** Cornelia de Lange Syndrome (CdLS), Whole exome sequencing, Novel variant, *NIPBL* gene, *SMC1A* gene, Mosaicism, Exome-negative

## Abstract

**Background:**

Cornelia de Lange Syndrome (CdLS) is a rare genetic disorder characterized by a range of physical, cognitive, and behavioral abnormalities. This study aimed to perform a comprehensive review of the literature on CdLS and investigate two cases of CdLS with distinct phenotypes that underwent WES to aid in their diagnosis.

**Methods:**

We conducted a comprehensive review of the literature on CdLS along with performing whole-exome sequencing on two CdLS patients with distinct phenotypes, followed by Sanger sequencing validation and in-silico analysis.

**Results:**

The first case exhibited a classic CdLS phenotype, but the initial WES analysis of blood-derived DNA failed to identify any mutations in CdLS-related genes. However, a subsequent WES analysis of skin-derived DNA revealed a novel heterozygous mutation in the *NIPBL* gene (NM_133433.4:c.6534_6535del, p.Met2178Ilefs*8). The second case was presented with a non-classic CdLS phenotype, and WES analysis of blood-derived DNA identified a heterozygous missense variant in the *SMC1A* gene (NM_006306.4:c.2320G>A, p.Asp774Asn).

**Conclusions:**

The study shows the importance of considering mosaicism in classic CdLS cases and the value of WES for identifying genetic defects. These findings contribute to our understanding of CdLS genetics and underscore the need for comprehensive genetic testing to enhance the diagnosis and management of CdLS patients.

## Introduction

Cornelia de Lange Syndrome (CdLS), also known as Brachmann-de Lange Syndrome, is a genetically and clinically heterogeneous disorder that affects multiple aspects of development and has a wide clinical variability [1, 2]. Over time, different diagnostic criteria have been proposed, and the disease has been classified as a spectrum (CdLS) based on a detailed diagnostic algorithm that combines “cardinal” and “suggestive” features. CdLS belongs to a broader group of conditions called cohesinopathies, which include patients carrying variants in one of the different genes belonging to the cohesin complex. The natural history of the disease includes multiple chronic medical problems, and several behavioral comorbidities have also been described [3]. CdLS is a rare condition, with an estimated incidence ranging from 1 in 10,000 to 1 in 30,000 newborns [3].

While the majority of CdLS cases appear to be sporadic, a few familial cases have been reported. Pedigree analyses of several families indicate autosomal dominant inheritance, with transmission observed from both parents in some cases [4]; however, given autosomal dominant inheritance, cases of apparently normal parents who have multiple children with CdLS were hypothesized to be the result of germline mosaicism that is one of the critical areas of medical genetics [5].

CdLS is associated with mutations in six genes of the cohesin complex, including *NIPBL*, *SMC1A*, *SMC3*, *RAD21*, *BRD4*, and *HDAC8* [6]. Most CdLS patients exhibit *de novo* pathogenic variants in one of these genes, with *NIPBL* being the most commonly affected gene [3]. The proteins encoded by these genes serve as structural or regulatory components of the cohesin complex, which is a multimeric system that regulates sister chromatid cohesion and gene expression. The core of the cohesin complex is composed of *SMC1A*, *SMC3*, *RAD21*, and *STAG*, and is involved in sister chromatid cohesion and gene expression regulation [7, 8].

The objective of this study is to present two novel heterozygous mutations identified in two patients with CdLS symptoms from unrelated families. These mutations contribute to the growing body of knowledge surrounding the genetic landscape of CdLS. Additionally, the study aims to underscore the importance of investigating mosaicism in patients exhibiting CdLS phenotypes. By identifying these mutations and highlighting the role of mosaicism, this research has implications for genetic counseling and clinical management of CdLS patients and their families.

## Case presentation

### Case 1

The research subject in question is a thirteen-year-old female, born to non-consanguineous parents. Her birth occurred at 37 weeks of gestational age via cesarean section, with recorded measurements of 2950 g in weight, 48 cm in height, and a head circumference of 32 cm (Table 1). No evidence indicating maternal infection or exposure during gestation has been identified in parental interviews or documents pertaining to the pregnancy period. Both parents displayed no clinical symptoms and had no notable family history. The patient exhibited various physical characteristics at birth, including a cleft palate, long philtrum, anteverted nares, low-set ears, long curly eyelashes, a short neck, and microcephaly. At the age of three and a half, she experienced her first seizure, which persisted as uncontrolled seizures for a duration of approximately ten years. As a result, her medication regimen underwent multiple changes, with her current treatment consisting of phenobarbital and sodium valproate. Furthermore, the patient received a diagnosis of intellectual disability and displayed developmental delays, rendering her unable to walk independently even at the age of thirteen, as she could only crawl. Since birth, she has faced challenges with feeding, constipation, and severe gastroesophageal reflux. Notably, her physical examination did not reveal any abnormalities in the extremities, ruling out upper limb reduction defects. Additionally, her speech development was delayed, and she could only articulate simple words at the age of thirteen. Laboratory analysis, including complete blood count, biochemical parameters, and urinalysis, yielded normal results. A transthoracic echocardiography examination identified a mild ventricular septal defect. Chromosome analysis confirmed a normal karyotype of 46, XX.

**Table 1:** Signs and symptoms CdLS and clinical finding of patients in this study

Signs and symptoms CdLS			Case 1	Case 2
Growth	IUGR	Suggestive	−	−
	Short stature	Suggestive	−	+
	Microcephaly	Suggestive	+	+
Craniofacial features	Brachycephaly	Suggestive	−	−
	Low anterior hairline	Other clinical Findings	−	+
	Arched, thick eyebrows	Cardinal	−	−
	Synophrys	Cardinal	−	+
	Long eyelashes	Other clinical Findings	+	+
	Depressed nasal bridge	Cardinal	−	+
	Anteverted nostrils	Cardinal	+	−
	Broad nasal tip	Cardinal	−	−
	Long, smooth philtrum	Cardinal	+	+
	Thin upper vermilion	Cardinal	−	+
	Downturned corners of the mouth	Cardinal	−	−
	Highly arched palate	Other clinical Findings	−	+
	Widely spaced teeth	Other clinical Findings	−	−
	Micrognathia	Other clinical Findings	−	+
	Low-set and malformed ears	Other clinical Findings	+	−
Trunk and limbs	Oligodactyly and adactyly (hands)	Cardinal	−	−
	Small hands	Suggestive	−	−
	Proximally placed thumbs	Other clinical Findings	−	−
	Clinodactyly or short fifth finger	Suggestive	−	−
	Small feet	Suggestive	−	−
	Hirsutism	Suggestive	−	−
	Cardiovascular anomalies	Other clinical Findings	+	+
	Vertebral anomalies	Other clinical Findings	−	−
	Congenital diaphragmatic hernia	Cardinal	−	−
Cognition and behaviour	Intellectual disability (any degree)	Suggestive	+	−
	ASD	Other clinical Findings	−	−
	Self-injurious behavior	Other clinical Findings	−	−
	Stereotypic movements	Other clinical Findings	−	−

ASD, autism spectrum disorder; IUGR, intrauterine growth retardation

### Case 2

Patient 2 was a nine-year-old girl born to parents who were not closely related. The pregnancy lasted approximately 36 weeks, resulting in a birth weight of 2300 g and a head circumference of 29 cm (Table 1). The delivery was performed via cesarean section. Throughout the pregnancy, no medications were administered, and there was no exposure to cigarettes or alcohol. The patient exhibited classic craniofacial features, including micrognathia, synophrys, microcephaly, low hairline, long curly eyelashes, thin upper lips, long philtrum, depressed nasal bridge, and high arched palate. Additionally, she presented with syndactyly of the second and third toes, small hands with the thumb positioned closer to the body, and a simian crease. A transthoracic echocardiography examination revealed a mild ventricular septal defect, but no abnormalities were found in her gastroesophageal function. An electroencephalogram showed no abnormalities, and she had no history of seizures. The patient experienced failure to thrive and developmental delay. She did not begin walking until the age of four, with the assistance of occupational therapy. Her speech development was delayed, and at the age of nine, she could only articulate simple words. Chromosome analysis revealed a normal karyotype of 46, XX.

## Methods

### Sample collection and genomic analysis

The genomic DNA was isolated from the peripheral blood of probands and their parent utilizing the salting-out procedure. The concentration and purity of DNA were ascertained using a NanoDrop 1000 (Thermo Fisher Scientific, Inc., Wilmington, DE, USA). Whole-Exome Sequencing **(**WES) was performed on an Illumina HiSeq4000 system with paired-end reads of 101 bp and 100X coverage, using genomic DNA from probands. Exonic and surrounding exon-intron border regions were enriched using SureSelectXT2 V6 kits.

Subsequent to the removal of low-quality reads, the reads were mapped to the human genome reference (hg19 build) with the aid of the Burrows-Wheeler Aligner (BWA). Duplicates were marked and removed using SAM tools. Following that, recalibration and SNP/indel calling were carried out. Variant calling and filtering were performed using the Genome Analysis Toolkit (GATK). Finally, the called variants were annotated, filtered, and prioritized using ANNOVAR software and an in-house workflow. The 3D protein structural model of mutations in *NIPBL* and *SMC1A* was modeled and visualized using PyMol software (The PyMOL Molecular Graphics System, Version 1.2r3pre, Schrödinger, LLC).

## Results

The homozygous variants identified in this study, which encompassed splicing regions, stop gain, and frameshift mutations, were primarily evaluated based on their degree of pathogenicity. Subsequently, the pathogenicity of nonsynonymous mutations was assessed using multiple amino acid change predictions. Emphasis was placed on prioritizing mutations in genes known to be associated with diseases exhibiting reported phenotypes.

During the WES analysis of case 1’s blood sample, no clinically significant variants related to the proband’s symptoms were found. However, the coding regions of *NIPBL*, *SMC1A*, *SMC3*, *HDAC8*, and *RAD21* genes related to CdLS received extra scrutiny due to a high clinical suspicion of the genetic syndrome. Therefore, WES of the skin sample was conducted, resulting in the detection of a heterozygous variant (NM_133433.4:c.6534_6535del (p.Met2178Ilefs*8) in the *NIPBL* found in 38% of reads. This mutation is novel and appears to exhibit a mosaic pattern in the proband as it was not detected in the previous analysis of blood-derived DNA. The variant entails a two-base pair deletion causing a frameshift in exon 38 and is absent from population databases such as Iranome and gnomAD [9, 10]. Based on the American College of Medical Genetics (ACMG) guidelines [11], this variant has been classified as a likely pathogenic variant.

Furthermore, a heterozygous variant in the *SMC1A* gene with dominant X-linked inheritance was primarily identified through WES in case 2. Subsequent confirmation was conducted using Sanger sequencing of genomic DNA. The analysis revealed that case 2 was heterozygous for the variant NM_006306.4: c.2320G > A (p.Asp774Asn), while both parents exhibited normal homozygosity. Similar to the *NIPBL* variant, the *SMC1A* variant was classified as a likely pathogenic variant in accordance with the guidelines provided by the American College of Medical Genetics (ACMG). This novel mutation, NM_006306.4: c.2320G > A, has not been documented in population databases, including gnomAD and Iranome. Furthermore, various prediction methods, such as MutationTaster, SIFT, polyphen, and UMD-predictor, have indicated its potential harmfulness [12, 13].

## Discussion

In this study, we performed a comprehensive narrative review of the literature on CdLS, with a focus on genotype-phenotype correlations. We also identified novel heterozygous mutations in two patients exhibiting CdLS manifestations, further expanding the genetic landscape of the disorder. Our analysis revealed that the mutations affect genes involved in chromatin regulation within the cohesin complex, which is consistent with previous research. Moreover, we underscored the importance of investigating mosaicism in CdLS cases, as one patient displayed mosaicism for a *NIPBL* mutation detected only in skin-derived DNA, but not in blood-derived DNA. Our findings demonstrate the value of genotype-phenotype correlations in understanding the clinical presentation of CdLS and improving diagnosis and management of affected individuals. By combining our novel findings with a comprehensive literature review, we provide valuable insights into the genetic basis of CdLS and its clinical manifestations.

The first description of CdLS was made by a Dutch pediatrician, Cornelia de Lange, in 1933 [14]. Over time, several authors have attempted to define diagnostic criteria, resulting in the identification of three clinical subtypes: classic, mild, and phenocopies [15]. The variability of clinical expression led in the past to clinical diagnosis only, but with the discovery of the complex and heterogeneous biological basis of the disease, molecular confirmation analysis became necessary. In 2018, a consensus statement was published, classifying CdLS as a spectrum (CdLSp) and providing a detailed diagnostic algorithm based on “cardinal” and “suggestive” features. The algorithm distinguishes between classic and non-classic CdLS, which do not overlap with the old definitions [3].

The natural history of the disease includes multiple chronic medical problems, pivotal for properly planning and addressing the follow-up of the affected patients. Several behavioral comorbidities have also been described. Additional cohesin- or chromatin-associated factors have also been identified, which are genetically different from cohesin but whose variants have been found in CdLS patients. The cohesin complex, in conjunction with the chromosome loader *NIPBL* and the sequence-specific DNA binding protein CTCF, organizes the genome into topologically associated domains (TADs), chromatin loops, and contact domains, which helps to orchestrate gene expression. Although CdLS cell lines do not display abnormalities in sister chromatid cohesion, they exhibit dysregulated genes and protein expression, suggesting that the disorder is due to altered transcriptional regulation resulting from an impaired function of the cohesin complex in 3D chromatin organization. Additionally, many genes have been found to be weakly transcriptionally dysregulated in CdLS animal models.

CdLS is classified into six types (MIM #300,590, #614,701, #300,882, #610,759, and #122,470) based on the specific genes involved in the cohesin complex, including *NIPBL, SMC1A, SMC3, RAD21, BRD4* and *HDAC8*. Additionally, mutations in *ANKRD11* genes have also been associated with CdLS [3]. The phenotypic spectrum of CdLS varies, with classic and non-classic forms observed. Classic CdLS is easily recognizable at birth due to distinctive craniofacial features, growth patterns, and limb defects. However, non-classic CdLS can present with different degrees of facial and limb involvement, making diagnosis more challenging [3].

CdLSp1, caused by mutations in the *NIPBL* gene, accounts for 70% of the cases and has been shown to cause both mild and severe forms of the syndrome. CdLSp2, associated with the *SMC1A* gene mutations, accounts for about 5% of cases. CdLSp3, CdLSp4, and CdLSp5 are caused by mutations in *SMC3*, *RAD21*, and *HDAC8*, respectively, and encompass the remaining CdLS cases. Most CdLSp1 patients with *NIPBL* mutations exhibit characteristic facial and skeletal changes, along with prenatal growth retardation, moderate to severe psychomotor deficiency, and major malformations leading to severe disability or death. However, it is important to note that individuals with variants in other causative CdLS genes can also meet the criteria for classic CdLS. Loss-of-function variants tend to result in more severe clinical features compared to missense variants, which are associated with a milder phenotype [4, 16–18].

Case 1 of this study also harbors a frameshift mutation, which is classified as a loss-of-function variant. This finding is consistent with previous reports indicating that severe clinical features are commonly associated with such mutations. Somatic mosaicism has been previously documented in CdLSp1 cases. Huisman et al. reported the detection of pathogenic *NIPBL* mutations in buccal cell-derived DNA from ten CdLSp1 patients whose blood-derived DNA analyses yielded negative results. Even upon resequencing of the *NIPBL* in the blood sample of those ten patients, the mutations could not be identified [19]. Along with previous studies, literature findings suggest that 15–20% of patients with classic CdLS phenotypes exhibit mosaicism with *NIPBL* mutations that are not detectable in blood-derived DNA [3]. Case 1 in this study presents characteristic clinical features, including a long philtrum, synophrys, depressed nasal bridge, thin upper lip, small hand, developmental delay, and microcephaly, which align with the established diagnostic criteria for classic CdLS as proposed by Kline et al. However, the epilepsy observed in Case 1 deviated from the reported cases of epilepsy in CdLS patients. Typically, partial epilepsy is the most prevalent type, with an age of onset typically before two years. In most reported instances, affected individuals respond favorably to standard medical therapy and can be successfully weaned off medication after a few years [3]. In contrast to these established patterns, the epilepsy in our reported case persisted as uncontrolled seizures despite the administration of various drugs. This departure from the expected form of epilepsy adds a distinctive aspect to our case and broaden the spectrum of epilepsy in the context of CdLS and necessitate further investigations. The consensus molecular diagnostic pathways for CdLS recommend initial screening using next-generation sequencing (NGS) for patients displaying classic phenotypes. If no variants in CdLS genes are identified, further investigation of mosaicism should be pursued by sequencing cells derived from tissues other than lymphocytes. In cases where such analysis yields no results, it is advisable to assess for deletions and duplications in the *NIPBL* [3]. In Case 1, the findings are consistent with previous reports where blood-derived DNA analysis failed to detect any CdLS-related mutations. However, a frameshift mutation was identified in the *NIPBL* when analyzing DNA derived from skin tissue. These observations highlight the prominence of mosaicism, particularly in patients exhibiting classical phenotypes of CdLS, and emphasize the importance of exploring alternative tissue sources if blood-derived DNA testing yields negative results. Mosaicism can occur due to various reasons, such as a mutation happening after fertilization, leading to some cells having the mutation while others do not. It can also occur during early embryonic development when cells divide and differentiate. The presence of mosaicism in CdLS patients with *NIPBL* mutations suggests that the mutation may have occurred at a later stage of development, resulting in a subset of cells having the mutation while others remain unaffected. This can lead to variation in the severity and presentation of CdLS symptoms among affected individuals. It is important to note that the exact reasons for mosaicism in CdLS patients with *NIPBL* mutations are still not fully understood and further research is needed to fully comprehend its implications on the disorder.

**Fig. 1:**
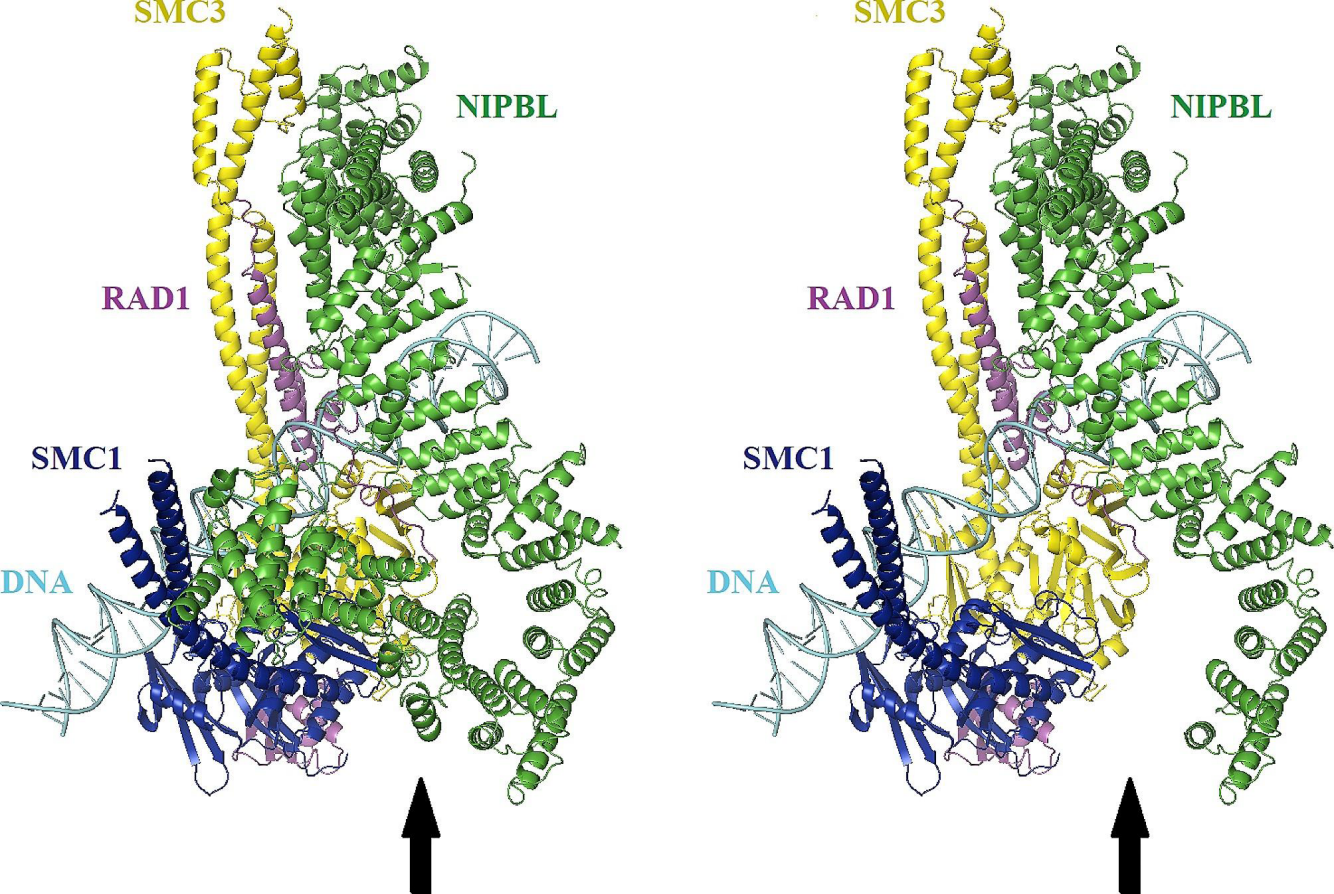
Overview of cohesin-NIPBL^C^ -DNA complex base on PDB 6WGE. The protein is coloured by protein. SMC1, SMC3, RAD21, NIPBL, and DNA are colored Blue, yellow, purple, green, and cyan, respectively. Left arrows show structure of normal human cohesin bound to the NIPBL^C^ and left arrows shows the structure of NIPBL^C^ with p.Met2178Ilefs*8 mutation in the complex. NIPBL^C=^ C-terminal HEAT repeat domain of NIPBL

**Fig. 2:**
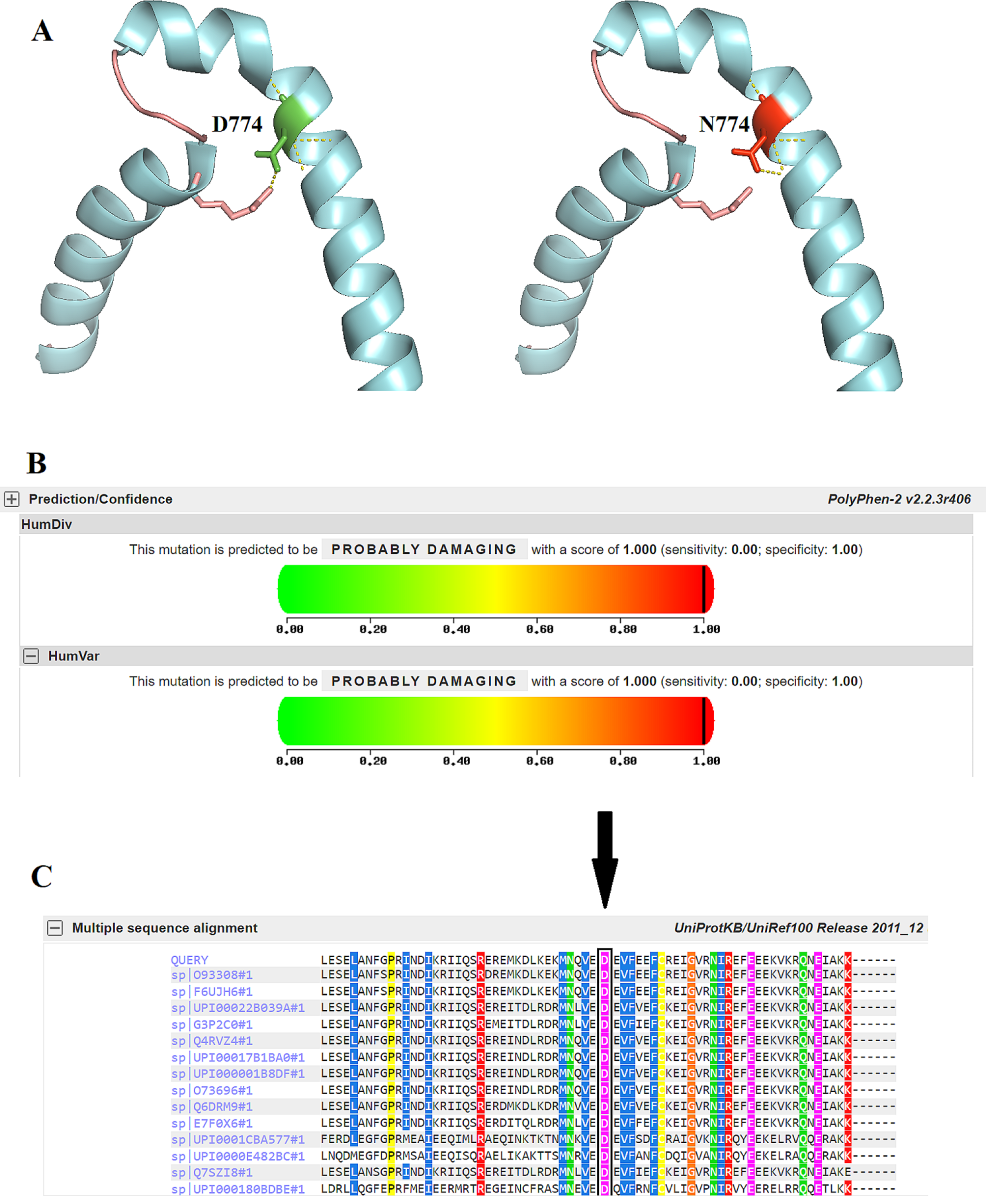
(**A**) Close-up of the wild type Asp(D) and mutated Asn(N) amino acid residues at position 774 in SMC1A. The side chain of the wild-type and the mutant residue are shown and coloured green and red respectively. (**B**) predicted score for D774N by polyphen-2 (**C**) Conservation of D774 among different species

CdLSp2, characterized by mutations in the *SMC1A* gene, is the second most common type of CdLS, accounting for approximately 5% of cases after CdLSp1 [20]. It has been observed that most CdLSp2 patients with *SMC1A* mutations are female, and the manifestations in females are attributed to the dominant-negative effect of the mutated *SMC1A* that escapes chromosome X-inactivation [21]. In contrast, males with *SMC1A* mutations typically exhibit a more severe phenotype compared to females. Initially, CdLSp2 was believed to be present with milder dysmorphic facial features, less affected growth patterns, and milder limb involvement compared to CdLSp1. However, there is variability in the phenotypic spectrum of *SMC1A*-related CdLS, and some cases have shown atypical features [22]. In this study, a patient (case 2) presented with several characteristic features of CdLS, such as a long philtrum, upturned nasal tip, thick eyebrows, microcephaly, and developmental delay. However, these features were classified as a non-classic phenotype based on the consensus clinical diagnostic criteria [3]. This finding is consistent with previous reports on CdLSp2 cases, which predominantly exhibit a non-classic phenotype. Based on the non-classic CdLS phenotypes observed in case 1, it is necessary to perform next-generation sequencing (NGS)-based screening of CdLS genes for accurate diagnosis of the patient. The WES analysis revealed the presence of the *SMCA1*:c.2320G > A (p.Asp774Asn) mutation, confirming the diagnosis of CdLS in the patient. In this mutation, the wild-type residue, Asp774, carries a negative charge, whereas the mutant residue, Asn774, carries a neutral charge. Consequently, the mutation results in the loss of charge in the wild-type residue, which may disrupt its interactions with other molecules or residues (Fig. 1). The wild-type residue is highly conserved, and the mutated residue is located within a domain crucial for binding other molecules (Fig. 2). Therefore, the mutation of this residue may interfere with its normal function. Numerous missense mutations in the *SMCA1* gene have been reported in association with CdLSp2, suggesting that missense mutations may represent a common disease mechanism. However, the *SMC1A*:c.2320G > A (p.Asp774Asn) mutation has not been reported in any previous cases. Future functional studies are necessary to elucidate the precise impact of the p. Asp774Asn mutation on SMCA1 function.

Furthermore, this study provides valuable insights into the phenotypic spectrum and diagnostic criteria of CdLS. It is characterized by a wide range of phenotypes, from classic to non-classic forms, which can complicate the diagnosis of the disease. The consensus criteria for CdLS diagnosis include cardinal and suggestive features, with a clinical score equal to or greater than 11 and three cardinal features confirming the diagnosis of classic CdLS. It is worth noting that different types of CdLS are associated with mutations in specific genes, such as CdLSp1 with *NIPBL* mutations and CdLSp2 with *SMC1A* mutations. However, there is variability in the phenotypic spectrum of CdLS, with some cases exhibiting milder dysmorphic features and less affected growth patterns. This study also emphasizes the importance of genetic testing, particularly NGS-based screening, for accurate diagnosis and management of CdLS patients, and highlights the need for further research to understand the functional consequences of novel variations in CdLS-associated genes.

Researchers have been attempting to identify correlations between molecular data and clinical phenotypes in CdLS since the discovery of new genes. However, the small number of patients with variants in genes other than *NIPBL* limits this effort. Variants in the *NIPBL* are responsible for the majority of classical and severe CdLS cases. Patients with missense variants have a better prognosis than those with loss of function variants. Variants in the *SMC1A*, *SMC3*, *RAD21*, *BRD4*, *ANKRD11* and *HDAC8* genes are rarer and have different phenotypic characteristics. Somatic mosaicism may contribute to less severe phenotypes, but further study is needed to determine its prevalence and consequences. CdLS-like phenotypes have also been observed in patients with variants in other genes associated with CdLS overlapping phenotypes.

## Conclusion

The study, conducted on CdLS, highlights the effectiveness of WES in identifying genetic defects that underlie unexplained neurological symptoms in affected individuals. The research expands upon the mutational spectrum of CdLSp1 and CdLSp2, emphasizing the importance of genetic testing for accurate diagnosis and management of patients. Furthermore, the study underscores the importance of mosaicism testing in patients with classic CdLS, whose blood-derived WES analysis may yield negative results for CdLS-related genes. These findings have important implications for genetic counseling and clinical management of CdLS patients and their families. By identifying these mutations and recognizing the role of mosaicism, healthcare professionals can provide more accurate diagnoses and appropriate treatment plans for CdLS patients. Further research is required to determine the functional consequences of the novel mutations identified in this study, which could provide additional insights into the relationship between mutation type and disease severity in CdLS. Overall, this study contributes valuable knowledge to the growing body of research on the genetic basis and clinical manifestation of CdLS, providing important insights for future research and clinical practice.

## Data Availability

The data and materials that support the findings of this study are available from the corresponding authors, upon request.

## Declarations

### ClinVar Accession

SCV001251685.1(*SMC1A*), SCV004099297(*NIPBL*).

## Abbreviations

CdLS: Cornelia de Lange Syndrome
BWA: Burrows-Wheeler Aligner
GATK: Genome Analysis Toolkit
ACMG: American College of Medical Genetics
TADs: topologically associated domains
NGS: next-generation sequencing

## References

[1] Dorsett D, Krantz ID (2009). On the molecular etiology of Cornelia De Lange syndrome. Ann N Y Acad Sci.

[2] Liu J, Krantz I (2009). Cornelia De Lange syndrome, cohesin, and beyond. Clin Genet.

[3] Kline AD, Moss JF, Selicorni A, Bisgaard A-M, Deardorff MA, Gillett PM (2018). Diagnosis and management of Cornelia De Lange syndrome: first international consensus statement. Nat Rev Genet.

[4] Gillis LA, McCallum J, Kaur M, DeScipio C, Yaeger D, Mariani A (2004). NIPBL mutational analysis in 120 individuals with Cornelia De Lange syndrome and evaluation of genotype-phenotype correlations. Am J Hum Genet.

[5] Krajewska-Walasek M, Chrzanowska K, Tylki‐Szymańska A, Bialecka M (1995). A further report of Brachmann‐De Lange syndrome in two sibs with normal parents. Clin Genet.

[6] Panaitescu AM, Duta S, Gica N, Botezatu R, Nedelea F, Peltecu G (2021). A broader perspective on the prenatal diagnosis of cornelia de lange syndrome: review of the literature and case presentation. Diagnostics.

[7] Haering CH, Schoffnegger D, Nishino T, Helmhart W, Nasmyth K, Löwe J (2004). Structure and stability of cohesin’s Smc1-kleisin interaction. Mol Cell.

[8] Avagliano L, Parenti I, Grazioli P, Di Fede E, Parodi C, Mariani M (2020). Chromatinopathies: a focus on Cornelia De Lange syndrome. Clin Genet.

[9] Lek M, Karczewski KJ, Minikel EV, Samocha KE, Banks E, Fennell T (2016). Analysis of protein-coding genetic variation in 60,706 humans. Nature.

[10] Chen S, Francioli LC, Goodrich JK, Collins RL, Kanai M, Wang Q et al. A genome-wide mutational constraint map quantified from variation in 76,156 human genomes. bioRxiv. 2022:2022.03. 20.485034.

[11] Green RC, Berg JS, Grody WW, Kalia SS, Korf BR, Martin CL (2013). ACMG recommendations for reporting of incidental findings in clinical exome and genome sequencing. Genet Sci.

[12] Schwarz JM, Rödelsperger C, Schuelke M, Seelow D (2010). MutationTaster evaluates disease-causing potential of sequence alterations. Nat Methods.

[13] Ng PC, Henikoff S (2003). SIFT: Predicting amino acid changes that affect protein function. Nucleic Acids Res.

[14] De Lange C (1933). Surun type nouveau degeneration (typus amestelodamensis). Arch Med Enfants.

[15] Selicorni A, Mariani M, Lettieri A, Massa V (2021). Cornelia De Lange syndrome: from a disease to a broader spectrum. Genes.

[16] Bhuiyan ZA, Klein M, Hammond P, van Haeringen A, Mannens MM, Van Berckelaer-Onnes I (2006). Genotype-phenotype correlations of 39 patients with Cornelia De Lange syndrome: the Dutch experience. J Med Genet.

[17] Selicorni A, Russo S, Gervasini C, Castronovo P, Milani D, Cavalleri F (2007). Clinical score of 62 Italian patients with Cornelia De Lange syndrome and correlations with the presence and type of NIPBL mutation. Clin Genet.

[18] Ansari M, Poke G, Ferry Q, Williamson K, Aldridge R, Meynert AM (2014). Genetic heterogeneity in Cornelia De Lange syndrome (CdLS) and CdLS-like phenotypes with observed and predicted levels of mosaicism. J Med Genet.

[19] Huisman SA, Redeker EJ, Maas SM, Mannens MM, Hennekam RC (2013). High rate of mosaicism in individuals with Cornelia De Lange syndrome. J Med Genet.

[20] Huisman S, Mulder PA, Redeker E, Bader I, Bisgaard AM, Brooks A (2017). Phenotypes and genotypes in individuals with SMC1A variants. Am J Med Genet Part A.

[21] Hoppman-Chaney N, Jang JS, Jen J, Babovic‐Vuksanovic D, Hodge JC (2012). In‐frame multi‐exon deletion of SMC1A in a severely affected female with Cornelia De Lange Syndrome. Am J Med Genet Part A.

[22] Huisman S, Mulder PA, Redeker E, Bader I, Bisgaard AM, Brooks A (2017). Phenotypes and genotypes in individuals with SMC1A variants. Am J Med Genet A.

